# 

**Published:** 1910-11

**Authors:** 


The Essentials of Histology, Descriptive and Practical. For the use of students. By Edward A. Schäfer, F.R.S., Professor of Physiology in the University of Edinburgh. New eighth edition, thoroughly revised. Octavo, 571 pages, with 645 illustrations. Lea & Febiger, Publishers, Philadelphia and New York, 1910. (Cloth, $3.50, net.)

Pathogenic Microörganisms, including Bacteria and Protozoa. A practical manual for students, physicians and health officers. By William H. Park, M.D., Professor of Bacteriology and Hygiene in the University and Bellevue Hospital Medical College, and Director of the Research Laboratory, Department of Health, New York City; and Anna W. Williams, M.D., Assistant Director of the Research Laboratory. Fourth edition, thoroughly revised. Octavo, 670 pages, with 196 illustrations and 8 full-page plates. Lea & Febiger, Publishers, Philadelphia and New York, 1910. (Cloth, $3.75, net.)

Obstetrical Nursing for Nurses and Students. By Henry Enos Tuley, A.M., M.D., Professor of Obstetrics, Medical Department University of Louisville. With seventy-three illustrations. Seond edition. John P. Morton & Company, Publishers, Louisville, Ky., 1910. (Cloth, $1.50.)

The Practice of Medicine. A guide to the nature, discrimination and management of disease. By A. O. J. Kelly, M.D., Assistant Professor of Medicine, University of Pennsylvania. Octavo, 949 pages, illustrated. Lea & Febiger, Publishers, Philadelphia and New York, 1910. (Cloth, $4.75, net.)

International Clinics. A Quarterly of Illustrated Clinical Lectures and especially prepared articles on Treatment, Medicine, Surgery, Neurology, Pediatrics, Obstetrics, Gynecology, Orthopedics, Pathology, Dermatology, Ophthalmology, Otology, Rhinology, Laryngology, Hygiene, etc. Edited by Henry W. Cattell, M.D. Vol. III, twentieth series. Philadelphia and London: J. B. Lippincott Co. 1910. (Cloth, $2.00.)

A Treatise on Orthopedic Surgery. By Royal Whitman, M.D., Adjunct Professor of Orthopedic Surgery in the College of Physicians and Surgeons, New York; Professor of Orthopedic Surgery in the New York Polyclinic. Fourth edition, revised and enlarged. Octavo, 908 pages, with 601 illustrations, mostly original. Lea & Febiger, Publishers, Philadelphia and New York. 1910. (Cloth, $5.50, net.)

The Practice of Surgery. By James Gregory Mumford, M.D., Visiting Surgeon to the Massachusetts General Hospital; Instructor in Surgery in the Harvard Medical School. Octavo, pp. 1015. With 682 illustrations. Philadelphia and London: W. B. Saunders Co. 1910. (Cloth, $7.00.)

The Practical Medicine Series. Ten volumes. Under the general editorial charge of Gustavus P. Head, M.D. Vol. V. Obstetrics. Edited by Joseph B. DeLee; M.D. Vol. VI. General Medicine. Edited by Frank Billings, M.D., and J. H Salisbury, M.D. Series 1910. Chicago: The Year Book Publishers. (Prices, $1.25 and $1.50; entire series, $10.00.)

Manual of Physiology with Practical Exercises. By G. N. Stewart, M.D., Professor of Experimental Medicine in Western Reserve University, Cleveland. Sixth edition. Small octavo, pp. 1084. Illustrated. New York: William Wood & Co. 1910. (Cloth, $5.00.)

Biology, General and Medical. By Joseph McFarland, M.D., Professor of Pathology and Bacteriology in the Medico-Chirurgical College of Philadelphia. 12 mo, pp. 440. With 160 illustrations. Philadelphia and London: W. B. Saunders Co. 1910. (Cloth, $1.75.)

Practical Points in Nursing. By Emily A. M. Stoney. Fourth edition. 12 mo, pp. 495. Illustrated. Philadelphia and London: W. B. Saunders Co. 1910. (Cloth, $1.75.)

A Treatise on Diseases of the Eye. By John E. Weeks, M.D., Professor of Ophthalmology in the University and Bellevue Hospital Medical College, New York. Octavo, pp. 944, with 528 illustrations and 25 full-page plates. Lea & Febiger, Publishers, Philadelphia and New York, 1910. (Cloth, $6.00, net.

Lea's Series of Medical Epitomes. Edited by Victor C. Pedersen, M.D., New York. An Epitome of Hygiene and Public Health. By George M. Price, M.D., formerly Inspector New York State Tenement Commission, Medical Sanitary Inspector, New York Department of Health. 12 mo, 255 pages. Lea & Febiger, Publishers, Philadelphia and New York. 1910. (Cloth, $1.00, net.)

